# Prevalence and Related Factors of White Coat Hypertension and Masked Hypertension in Shunde District, Southern China

**DOI:** 10.3389/fphys.2022.936750

**Published:** 2022-07-01

**Authors:** Hailan Zhu, Haoxiao Zheng, Xiaoyan Liang, Chunyi Huang, Lichang Sun, Xiong Liu, Min Qiu, Weiyi Mai, Yuli Huang

**Affiliations:** ^1^ Department of Cardiology, Shunde Hospital, Southern Medical University, Foshan, China; ^2^ Department of Health Check-up Centre, Shunde Hospital, Southern Medical University, Foshan, China; ^3^ Department of Cardiology, The First Affiliated Hospital of Sun Yat-sen University, Guangzhou, China; ^4^ The George Institute for Global Health, newtown, NSW, Australia

**Keywords:** white-coat hypertension, masked hypertension, risk factors, prevalence, home blood pressure monitoring, telemedicine

## Abstract

**Background:** White coat hypertension (WCH) and masked hypertension (MH) can increase the risk of target organ damage. Home blood pressure monitoring is an important method for detecting WCH and MH. However, the prevalence and related factors of WCH and MH in China have been rarely reported.

**Objective:** To explore the prevalence and related factors associated with white coat hypertension (WCH) and masked hypertension (MH) in Shunde District, Southern China.

**Methods:** This study recruited subjects from the Physical Examination Center in Shunde Hospital, Southern Medical University. Office blood pressure and home blood pressure values were collected using the home blood pressure monitor with telemedicine device and office blood pressure monitor, and the prevalence of WCH and MH was calculated by the values. Multivariate logistic regression was used to explore the related factors for WCH and MH.

**Results:** Four-hundred and sixty-one participants (61% male), with an average age of 49 years, were included. The prevalence of WCH and MH was 5.1 and 15.2%, respectively. Multivariate logistic regression analysis showed that smoking (OR = 4.71, 95% CI = 1.05–21.15) and family history of coronary heart disease (OR = 4.51, 95% CI = 1.08–18.93) were associated with higher odds of WCH. The associated factors for higher odds of MH were smoking (OR = 2.83, 95% CI = 1.11–7.23), family history of hypertension (OR = 2.17, 95% CI = 1.11–4.26) and family history of coronary heart disease (OR = 2.82, 95% CI = 1.07–7.45).

**Conclusion:** WCH and MH are highly prevalent in the Physical Examination Center in Shunde Hospital, Southern Medical University. We found smoking and family history of coronary heart disease were related factors for WCH, and smoking, family history of hypertension and coronary heart disease were associated with the odds of MH. Home blood pressure monitoring with a telemedicine device should be recommended to identity abnormal BP phenotype.

## Introduction

Hypertension is one of the most common chronic diseases and causes a serious public health burden worldwide (Forouzanfar et al., 2017; Liu et al., 2019). To improve the management of hypertension, most academic guidelines stated that out-of-office blood pressure (BP) monitoring should be recommended in high-risk populations (Whelton et al., 2018; Williams et al., 2018; Liu et al., 2019). By combining the values of office BP and out-of-office BP monitoring, two specific abnormal BP phenotypes, masked hypertension (MH) and white coat hypertension (WCH) were proposed (Zhu et al., 2020a). MH is defined as an elevated out-of-office BP but normal in-office BP, which has been generally considered harmful and require appropriate treatment (Banegas et al., 2018; Fujiwara et al., 2018). In contrast to MH, WCH refers to individuals with an elevated office BP but normal out-of-office BP. WCH had been considered as a ‘benign’ phenomenon (Briasoulis et al., 2016; Myers and Stergiou, 2016). However, our recent study showed that WCH was also associated with a higher risk of cardiovascular events and all-cause death compared with normal BP (Huang et al., 2017). Therefore, early detection and management of MH and WCH would be of important clinical significance. Studies reported that the overall prevalence of WCH in the general population is 9–23% and the prevalence of MH ranges from 6.7 to 20% in different reports (Zhu et al., 2020a). Hypertension is highly prevalent in China, however, the prevalence of WCH and MH, as well as their associated factors in China have been rarely reported.

Hence, we conduct a cross-sectional study to explore the prevalence and related factors of WCH and MH in health check-up center in Shunde District, Southern China, using data from community-based health check-up information and the remote intelligent home BP monitoring platform.

## Methods

### Study Population and Design

The HBPM-iCloud (Home Blood Pressure Monitoring Cohort Study Based on Remote Intelligent Cloud Platform) study is an open prospective cohort study, recruiting participants from health checkup centers in Southern China. The current cross-sectional analysis was part of the cohort study. Detailed methods have been previously described (Zhu et al., 2020b). In brief, recruitment began in January 2019, and we recruited the general population attending health check-up examinations in Shunde Hospital of Southern Medical University. Adults aged 18 years or older who provide written informed consent were screened. Those who were 1) unable to conduct BP by themselves, 2) diagnosed with secondary hypertension previously, 3) without physical examination data, 4) with less than 2 days of home BP data, or 5) have a history of malignancy would be excluded. After enrollment, a unified validated upper-arm electronic HBPM device (pulse wave BP-88G, Raycome, Shenzhen, China) would be provided to participants for free use over the following week. The prevalence of different BP phenotypes was calculated based on the office BP and home BP values.

This project has been approved by the ethics committee of Shunde Hospital of Southern Medical University. All participants gave written informed consent.

### Data Collection

Demographic information and medical history were collected including the following items: gender, age, smoking history (refers to smoking at least 1 cigarette per day for the past 6 months), alcohol consumption history (refers to drinking wine at least once a week in the past year), height, weight, employment in shift work, physical activity status (refers to exercising at least twice a week in the past year), family history of hypertension/coronary heart disease/stroke, and personal history of hypertension/cardiovascular diseases/other diseases. Body mass index (BMI) was calculated as weight (kg) divided by height (m) squared, and classified with the following categories: underweight (<18.5 kg/m^2^), normal weight (18.5–23.9 kg/m^2^), overweight (24.0–27.9 kg/m^2^), and obese (≥28.0 kg/m^2^) (Huang et al., 2014a). Routine physical examinations including blood biochemical items were also recorded.

### Office Blood Pressure and Home Blood Pressure Monitoring

The office BP was measured by a trained physician using a validated automatic upper-arm device (OMRON HEM-7118, Japan) according to the 2018 Chinese Guidelines for The Prevention and Treatment of Hypertension (1). Participants should rest for at least 5 mins and be in a straight sitting position with measured upper arm exposed, keeping the elbow at the same level with the heart. The average of two measurements was recorded as the office BP. At the first visit, both upper arms would be measured and the one with higher reading would be recorded as the preferred measured arm. The home BP was measured following the 2019 Chinese guidelines for Home Blood Pressure Monitoring (Wang et al., 2020). Before self-measurement, participants were asked to rest in a relaxed and comfortable position for at least 5 mins. Measurements were taken twice every morning and evening for seven consecutive days. The average of the last 6 days readings was recorded as home BP. Considering the fact that some participants may have poor compliance and cannot measure for 7 consecutive days, we divided the participants into two groups: one with measurements < 4 consecutive days, and the other with ≥ 4 consecutive days.

### BP Phenotypes Classifications

**FIGURE 1:**
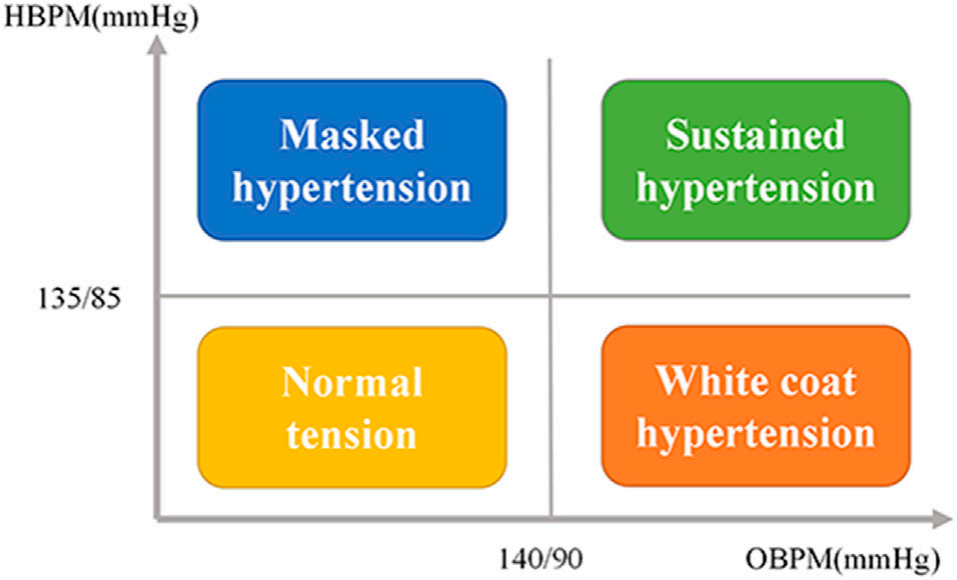
The classification of BP subtypes by combination of office and home BP readings. BPM = Home blood pressure monitoring; OBPM = Office blood pressure monitoring; BP = Blood pressure.

According to the 2018 Chinese Guidelines for The Prevention and Treatment of Hypertension (1), in our current study, hypertension was defined as patients with office BP ≥ 140/90 mmHg or with average home BP ≥ 135/85 mmHg or with a history of hypertension and taking anti-hypertensive medication currently. We further divided participants without anti-hypertensive medications into the following four groups: 1) Normotension (NT): The office BP was <140/90 mmHg, and the home BP was <135/85 mmHg; 2)White coat hypertension (WCH): The office BP was ≥140/90 mmHg, and home BP was <135/85 mmHg; 3) Masked hypertension (MH): office BP was <140/90 mmHg, and home BP was ≥135/85 mmHg; 4) Sustained hyperetension (SH): office BP was ≥140/90 mmHg, and home BP was ≥135/85 mmHg (Figure 1) (5).

### Data Management and Analysis

To investigate the prevalence of WCH and MH in the health check-up center of Shunde Hospital, Southern Medical University, we estimated the sample size based on a previous study (Zhang et al., 2015) (in which the prevalence of WCH and MH were 10.3 and 20%, respectively), using two-tailed hypotheses for 90% study power to detect a type I error at 0.05, with the possibility of 20% dropout rate. Finally, a sample size of at least 330 cases was recommended. Continuous data were expressed as mean ± standard deviation and compared using the ANOVA test. Categorical data were expressed as percentages and compared using a Chi-square test. Multivariate logistic regression analysis was used to investigate the related factors of different BP phenotypes, with the NT group as reference. Statistical analysis was performed using SPSS 20.0 (IBM Corporation, Armonk, New York, United States). All statistical tests were two-sided and a *p* value < 0.05 was considered statistically significant.

## Results

### The Prevalence of WCH and MH in the Health Check-Up Population

From January 2019 to December 2020, 594 participants were recruited in the study. We excluded 58 participants with secondary hypertension, 6 with tumors, 38 participants with less than 2 days of home BP data, and 31 participants without adequate physical examination data. Finally, 461 participants (61% male) were included in the analysis, with an average age of 49 years. The mean number of measure days was 5 days, with mean measure times of 4 per day.

**FIGURE 2:**
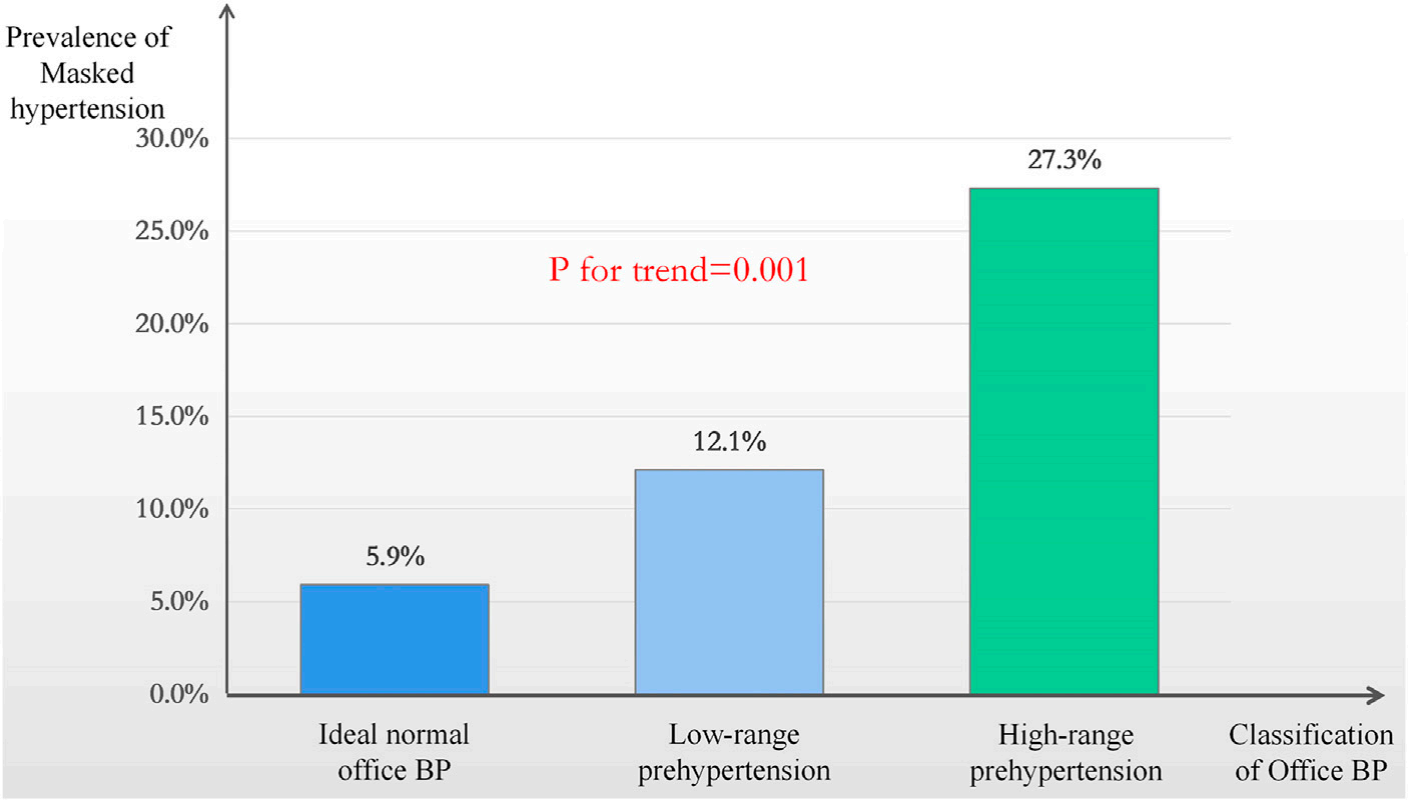
The prevalence of masked hypertension in different office blood pressure distribution. BP = Blood pressure.

In the 374 participants without anti-hypertensive medications, the prevalence of NT, WCH, MH, and SH was 56.40% (211 cases), 5.10% (19 cases), 15.20% (57 cases) and 23.30% (87 cases), respectively. The clinical baseline data were shown in Table 1. Most notably, we found that the systolic BP and diastolic BP in the MH group were both higher than those in the NT group (131.86 ± 6.73 mmHg vs 127.23 ± 10.88 mmHg, 84.42 ± 5.30 mmHg vs 80.11 ± 7.39 mmHg, respectively, with *p* < 0.05 for both groups after the Bonferroni method correction, Table 1). We then divided the office BP into the following three categories: ideal normal office BP (<120/80 mmHg), low-range prehypertension (120-129/80-84 mmHg) and high-range prehypertension (130-139/85-89 mmHg). Further analysis showed that the prevalence of MH in the above-mentioned subgroups was 5.90, 12.10 and 27.30%, respectively, (*p* for trend = 0.001, Figure 2).

**TABLE 1:** Comparison of baseline clinical data under different blood pressure types.

Variables	NT (*n* = 211)	WCH (*n* = 19)	MH (*n* = 57)	SH (*n* = 87)	*p*
Age, y	45.73 ± 13.43	50.89 ± 12.46	46.63 ± 12.10	48.34 ± 12.73	0.212
Male, n (%)	119 (56.4)	10 (52.6)	38 (66.7)	66 (75.9)	0.010
Overweight or obese, n (%)	99 (48.1)	7 (36.8)	38 (66.7)	56 (65.1)	0.004
Heart rate, n/min	73.33 ± 10.27	76.18 ± 9.54	76.02 ± 9.32	76.52 ± 10.39	0.047
Mean OSBP(mmHg)	127.23 ± 10.88^a^	152.63 ± 9.06	131.86 ± 6.73^a^	152.90 ± 11.68	<0.001
Mean ODBP(mmHg)	80.11 ± 7.39^b^	93.61 ± 8.31	84.42 ± 5.30^b^	98.97 ± 9.61	<0.001
Mean HSBP(mmHg)	123.50 ± 9.26^a^	130.85 ± 3.51^a^	138.40 ± 8.14	147.51 ± 11.61	<0.001
Mean HDBP(mmHg)	78.46 ± 6.40^b^	82.18 ± 3.69^b^	92.49 ± 5.65	98.06 ± 8.72	<0.001
Smoke, n (%)	20 (11.0)	6 (31.6)	18 (32.1)	27 (31.8)	<0.001
Drink, n (%)	67 (36.8)	7 (36.8)	27 (48.2)	55 (64.7)	<0.001
Family history of hypertension, n (%)	62 (34.3)	5 (26.3)	32 (57.1)	56 (66.7)	<0.001
Family history of CHD, n (%)	12 (6.7)	4 (21.1)	12 (21.4)	15 (17.6)	0.006
Family history of stoke, n (%)	15 (8.2)	2 (10.5)	7 (12.5)	13 (15.3)	0.362
Family history of early CHD, n (%)	7 (3.8)	0 (0)	2 (3.6)	4 (4.7)	0.965
Personal history of CHD/stroke, n (%)	2 (1.1)	1 (5.3)	2 (3.6)	1 (1.2)	0.223
Personal history of diabetes, n (%)	8 (4.4)	1 (5.3)	6 (10.7)	3 (3.5)	0.363
Shift work, n (%)	19 (10.4)	3 (15.8)	9 (16.1)	12 (14.1)	0.630
Measure days >4, n (%)	160 (75.8)	16 (84.2)	51 (89.5)	83 (95.4)	<0.001
Exercise, n (%)	56 (30.8)	4 (21.1)	10 (17.9)	8 (9.4)	0.001
TC (mmol/l)	5.44 ± 1.01	5.72 ± 1.55	5.48 ± 1.15	5.73 ± 1.19	0.258
LDL-C (mmol/l)	2.94 ± 0.74	3.24 ± 1.19	3.02 ± 0.80	3.13 ± 0.79	0.230
HDL-C (mmol/l)	1.51 ± 0.43	1.51 ± 0.43	1.38 ± 0.33	1.48 ± 0.41	0.315
TG (mmol/l)	1.56 ± 0.94	1.30 ± 0.39	1.95 ± 1.24	1.81 ± 1.30	0.062
ALT (U/L)	32.03 ± 28.61	25.93 ± 18.98	32.77 ± 19.25	35.30 ± 27.45	0.616
AST (U/L)	23.77 ± 14.15	22.86 ± 10.33	26.45 ± 15.46	23.44 ± 8.53	0.572
HCY(μmol/l)	9.01 ± 1.90	11.11 ± 3.38	10.38 ± 2.01	10.46 ± 2.87	0.014
CRE(μmol/l)	73.18 ± 15.43	68.78 ± 15.35	75.06 ± 13.50	80.22 ± 18.62	0.006
e-GFR (ml/min)	108.82 ± 23.10	110.74 ± 23.81	104.81 ± 20.86	100.27 ± 25.71	0.056

a The total difference between the four groups was *p* < 0.001, and the pairwise comparison between groups with * was statistically significant, after further corrected by Bonferroni method, with *p* = 0.042.

b The total difference between the four groups was *p* < 0.001, and the pairwise comparison between groups with † was statistically significant, after further corrected by Bonferroni method, with *p* < 0.001.

Abbreviations: NT = Normal tension; WCH = White coat hypertension; MH = Masked hypertension; SH = Sustained hypertension; OSBP = Office systolic blood pressure; ODBP = Office diastolic blood pressure; CHD = Chronic heart disease; C = Total cholesterol; LDL-C = Low density lipid-cholesterol; HDL-C=High density lipid-cholesterol; TG = Total triglycerides; ALT = Alanine transaminase; AST = Aspartate aminotransferase; HCY = homocysteine; CRE = Creatinine; e-GFR = estimated-Glomerular Filtration Rate.

### Related Factors For WCH and MH

To explore the related factors of WCH and MH, we finally included age, sex, smoking history, alcohol consumption history, BMI, physical activity status, resting heart rate, family history of hypertension, family history of coronary heart disease and measurement days in the logistic regression model, most of which were statistically significant in the clinical baseline data comparison. The multivariate logistic regression showed that smoking (OR = 4.71, 95% CI = 1.05–21.15, *p* = 0.043) and family history of coronary heart disease (OR = 4.51, 95% CI = 1.08–18.93, *p* = 0.039) were the independent related factors for WCH (Table 2), while smoking (OR = 2.83, 95% CI = 1.11–7.23, *p* = 0.03), family history of hypertension (OR = 2.17, 95% CI = 1.11–4.26, *p* = 0.024) and family history of coronary heart disease (OR = 2.82, 95% CI = 1.07–7.45, *p* = 0.036) were the independent related factors for MH (Table 3).

**TABLE 2:** Multivariate logistic regression analysis for related factors in White coat hypertension.

Variables	OR	95% CI	*p*
Male	1.05	0.27-4.07	0.947
Smoking history	4.71	1.05-21.15	0.043
Family history of hypertension	0.46	0.14-1.51	0.200
Family history of CHD	4.51	1.08-18.93	0.039
Exercise	0.65	0.19-2.18	0.480
Age	1.03	0.99-1.07	0.170
Overweight or obese	0.53	0.19-1.51	0.235
Drinking history	0.69	0.19-2.50	0.571
Measurement days >4	1.55	0.34-7.03	0.572
Heart rates	1.03	0.97-1.08	0.174

CHD, chronic heart disease.

**TABLE 3:** Multivariate logistic regression analysis for related factors in Masked hypertension.

Variables	OR	95% CI	*p*
Male	0.60	0.25-1.43	0.247
Smoking history	2.83	1.11-7.23	0.030
Family history of hypertension	2.17	1.11-4.26	0.024
Family history of CHD	2.82	1.07-7.45	0.036
Exercise	0.74	0.33-1.67	0.468
Age	1.01	0.97-1.04	0.337
Overweight or obese	1.77	0.89-3.53	0.104
Drinking history	0.79	0.36-1.75	0.557
Measurement days >4	2.05	0.67-6.32	0.211
Heart rates	1.03	0.99-1.06	0.134

CHD, chronic heart disease.

## Discussion

### Principle Results

In this cross-sectional study, we found WCH and MH are highly prevalent in the health check-up population in Shunde district, Southern China. Individuals with WCH presented with related factors as smoking and family history of coronary heart disease, while smoking, family history of hypertension and family history of coronary heart disease were the independent related factors for MH.

### Comparison With Prior Work

Studies have shown that MH and WCH can increase the risk of target organ damage (Palla et al., 2018; Cohen et al., 2019). In our investigation, the prevalence of WCH and MH was 5.10 and 15.20%, respectively, which suggests that in the study population, using office BP alone as the diagnostic and exclusive criteria for hypertension would result in 5.10% of WCH and 15.20% of MH patients being misdiagnosed. Therefore, it is necessary to promote the use of out-of-office BP monitoring to screen for WCH and MH in the physical examination population. What’s more, from the baseline data we found that the systolic BP and diastolic BP in the MH group were both higher than those in the NT group. Further investigation showed that in the higher range prehypertension group, the prevalence of MH was up to 27.30%, significantly higher than that of the ideal office BP group. These results were consistent with the previous study (Niiranen et al., 2012), suggesting that patients with higher office BP should pay close attention to the possibility of MH. Thus, the use of out of office BP monitoring should be more promoted to the pre-hypertension population. It should be noted that our previous studies showed the increased risk of target organ damage in pre-hypertension (Huang et al., 2013; Huang et al., 2014b). Given the high prevalence of MH in pre-hypertension, it remains unclear whether target organ damage is caused by undetected MH or prehypertension (Chen et al., 2015) and thus requires further studies.

Primary hypertension is a multifactorial chronic disease, which is mainly affected by genetic and environmental factors and their interactions. The traditional related factors for hypertension include genetic factors and environmental factors (such as diet, mental stress and smoking, etc.). Previous studies found that age, sex, smoking history, drinking history, BMI, diabetes history and cardiovascular history of family members were related factors for MH (Wang et al., 2017; Kario et al., 2019; Trudel et al., 2019), and related factors for WCH included smoking history, alcohol consumption history, age and sex (Afsar, 2013; Omboni et al., 2016; Kario et al., 2019). In the current study, we found that smoking and family history of coronary heart disease were related factors for WCH. The related factors for MH were smoking, family history of hypertension and family history of coronary heart disease. It should be noted that compared with non-smokers, the prevalence of WCH and MH in smokers was 4.71 and 2.83 times, respectively. Previous available data also found the evidence on the association between smoking and hypertension (Bowman et al., 2007; Lee et al., 2017; Wu et al., 2017; Bernabe-Ortiz and Carrillo-Larco, 2021). A prospective cohort study including 28,236 participants showed that compared to non-smokers, those who smoked ≥25 cigarettes per day was associated with increased risk of hypertension (Bowman et al., 2007). Another national survey also found that smoking increased the risk of hypertension (Lee et al., 2017). The possible mechanisms may attribute to that smoking is involved in the vascular damage through increasing stiffening of arterial walls, oxidative stress, platelet stickiness and reactivity and damage to endothelium, thus increase the arterial blood pressure (Howard et al., 1994; Lee et al., 2017; Bernabe-Ortiz et al., 2021). Therefore, smoking cessation may help to reduce the occurrence and prevent the progression of WCH and MH.

### Implication For Research and Practice

Home BP monitoring is an important method for detecting WCH and MH. However, the situation of home BP monitoring in China is not as optimistic as expected. Recently, Zuo et al. investigated the use of Home BP monitoring among the hypertensive population in 20 Chinese communities (Zuo et al., 2020), which showed that among 2,272 hypertensive patients, only 45.3% had a home BP monitor, and only 16% actively reported home BP values to physicians, which suggested that the application of home BP monitoring in Chinese hypertensive population is less than satisfactory. In addition, recording bias and arbitrary self-modification of anti-hypertensive treatment by anxious patients are also common in routine home BP monitoring. In this current study, we used a home BP monitor with the remote intelligent data-transmission service, which can avoid the above shortcomings. These are the pivotal differences that distinguish our study from the J-HOP study (Kawauchi et al., 2018) and the Finn-home study (Niiranen et al., 2010). Many studies also showed that telemedicine can significantly improve the BP control in the hypertensive patients compared to conventional treatment (Margolis et al., 2013; Stoddart et al., 2013; Duan et al., 2017). Therefore, in the near future, telemedicine may play an important role in the management of hypertension. In order to better incorporate home BP monitoring into clinical practice, the following measures should be taken in addition to strengthening the propaganda and education of hypertension prevention and treatment: 1) Physicians need to be aware of the indications and limitations of home BP monitoring and recommend home BP monitoring for the appropriate population; 2) Patients should receive clearer training and education on home BP monitoring, including correct BP measurement methods and instrument calibration; 3) The telemonitoring of home BP monitoring should be taken into consideration for further improvement of BP management.

### Limitations

Several limitations should be noted in the current study. Firstly, participants were recruited from health check-up centers, but not a community population, which may result in an underlying bias in the results, which cannot be generalized to the national population. Secondly, it has been reported that both MH and WCH display poor reproducibility over time (Stenehjem and OS, 2004; Viera et al., 2014). Therefore, the short-term use of home BP monitoring in the current study may cause misclassification of BP phenotypes. Finally, compared to a previous multicenter study in China (Kang et al., 2015), the prevalence of white coat hypertension is relatively low in the current study. The relative small sample of our study may lead to false positive results with large confidence interval in Logistic regression analyses.

## Conclusion

WCH and MH are highly prevalent in the Physical Examination Center in Shunde Hospital, Southern Medical University. We found smoking and family history of coronary heart disease were related factors for WCH, and smoking, family history of hypertension and coronary heart disease were associated with higher odds of MH. Home blood pressure monitoring with a telemedicine device should be recommended to identity abnormal BP phenotype.

## Data Availability

The original contributions presented in the study are included in the article/Supplementary Material, further inquiries can be directed to the corresponding author.
