# Supplementary material for: Prevalence and Related Factors of White Coat Hypertension and Masked Hypertension in Shunde District, Southern China
Source: Front Physiol. 2022 Jul 1;13:936750. doi: 10.3389/fphys.2022.936750 (PMC9283833; doi:10.3389/fphys.2022.936750)
Supplement: Supplementary file 1 [file Table1.docx]

Table 1. Comparison of baseline clinical data under different blood pressure types

| Variables | NT(n=211) | WCH(n=19) | MH(n=57) | SH(n=87) | P |
| --- | --- | --- | --- | --- | --- |
| Age, y | 45.73±13.43 | 50.89±12.46 | 46.63±12.10 | 48.34±12.73 | 0.212 |
| Male, n(%) | 119(56.4) | 10(52.6) | 38(66.7) | 66(75.9) | 0.010 |
| Overweight or obese, n(%) | 99(48.1) | 7(36.8) | 38(66.7) | 56(65.1) | 0.004 |
| Heart rate, n/min | 73.33±10.27 | 76.18±9.54 | 76.02±9.32 | 76.52±10.39 | 0.047 |
| Mean OSBP(mmHg) | 127.23±10.88* | 152.63±9.06 | 131.86±6.73* | 152.90±11.68 | <0.001 |
| Mean ODBP(mmHg) | 80.11±7.39† | 93.61±8.31 | 84.42±5.30† | 98.97±9.61 | <0.001 |
| Mean HSBP(mmHg) | 123.50±9.26* | 130.85±3.51* | 138.40±8.14 | 147.51±11.61 | <0.001 |
| Mean HDBP(mmHg) | 78.46±6.40† | 82.18±3.69† | 92.49±5.65 | 98.06±8.72 | <0.001 |
| Smoke, n(%) | 20(11.0) | 6(31.6) | 18(32.1) | 27(31.8) | <0.001 |
| Drink, n(%) | 67(36.8) | 7(36.8) | 27(48.2) | 55(64.7) | <0.001 |
| Family history of hypertension, n(%) | 62(34.3) | 5(26.3) | 32(57.1) | 56(66.7) | <0.001 |
| Family history of CHD, n(%) | 12(6.7) | 4(21.1) | 12(21.4) | 15(17.6) | 0.006 |
| Family history of stoke, n(%) | 15(8.2) | 2(10.5) | 7(12.5) | 13(15.3) | 0.362 |
| Family history of early CHD, n(%) | 7(3.8) | 0(0) | 2(3.6) | 4(4.7) | 0.965 |
| Personal history of CHD/stroke, n(%) | 2(1.1) | 1(5.3) | 2(3.6) | 1(1.2) | 0.223 |
| Personal history of diabetes, n(%) | 8(4.4) | 1(5.3) | 6(10.7) | 3(3.5) | 0.363 |
| Shift work, n(%) | 19(10.4) | 3(15.8) | 9(16.1) | 12(14.1) | 0.630 |
| Measure days >4, n(%) | 160(75.8) | 16(84.2) | 51(89.5) | 83(95.4) | <0.001 |
| Exercise, n(%) | 56(30.8) | 4(21.1) | 10(17.9) | 8(9.4) | 0.001 |
| TC(mmol/l) | 5.44±1.01 | 5.72±1.55 | 5.48±1.15 | 5.73±1.19 | 0.258 |
| LDL-C(mmol/l) | 2.94±0.74 | 3.24±1.19 | 3.02±0.80 | 3.13±0.79 | 0.230 |
| HDL-C(mmol/l) | 1.51±0.43 | 1.51±0.43 | 1.38±0.33 | 1.48±0.41 | 0.315 |
| TG(mmol/l) | 1.56±0.94 | 1.30±0.39 | 1.95±1.24 | 1.81±1.30 | 0.062 |
| ALT(U/L) | 32.03±28.61 | 25.93±18.98 | 32.77±19.25 | 35.30±27.45 | 0.616 |
| AST(U/L) | 23.77±14.15 | 22.86±10.33 | 26.45±15.46 | 23.44±8.53 | 0.572 |
| HCY(μmol/l) | 9.01±1.90 | 11.11±3.38 | 10.38±2.01 | 10.46±2.87 | 0.014 |
| CRE(μmol/l) | 73.18±15.43 | 68.78±15.35 | 75.06±13.50 | 80.22±18.62 | 0.006 |
| e-GFR(ml/min) | 108.82±23.10 | 110.74±23.81 | 104.81±20.86 | 100.27±25.71 | 0.056 |

*The total difference between the four groups was P<0.001, and the pairwise comparison between groups with * was statistically significant, after further corrected by Bonferroni method, with P=0.042.

†The total difference between the four groups was P<0.001, and the pairwise comparison between groups with † was statistically significant, after further corrected by Bonferroni method, with P<0.001

**Abbreviations:** NT=Normal tension; WCH=White coat hypertension; MH=Masked hypertension; SH=Sustained hypertension; OSBP=Office systolic blood pressure; ODBP=Office diastolic blood pressure; CHD=Chronic heart disease; TC=Total cholesterol; LDL-C=Low density lipid-cholesterol; HDL-C=High density lipid-cholesterol; TG=Total  triglycerides; ALT=Alanine transaminase; AST=Aspartate aminotransferase; HCY=homocysteine; CRE=Creatinine; e-GFR=estimated-Glomerular Filtration Rate.
