# Supplementary material for: Prevalence and Related Factors of White Coat Hypertension and Masked Hypertension in Shunde District, Southern China
Source: Front Physiol. 2022 Jul 1;13:936750. doi: 10.3389/fphys.2022.936750 (PMC9283833; doi:10.3389/fphys.2022.936750)
Supplement: Supplementary file 2 [file Table2.docx]

Table 2. Multivariate logistic regression analysis for related factors in White coat hypertension.

| Variables | OR | 95%CI | P |
| --- | --- | --- | --- |
| Male | 1.05 | 0.27-4.07 | 0.947 |
| Smoking history | 4.71 | 1.05-21.15 | 0.043 |
| Family history of hypertension | 0.46 | 0.14-1.51 | 0.200 |
| Family history of CHD | 4.51 | 1.08-18.93 | 0.039 |
| Exercise | 0.65 | 0.19-2.18 | 0.480 |
| Age | 1.03 | 0.99-1.07 | 0.170 |
| Overweight or obese | 0.53 | 0.19-1.51 | 0.235 |
| Drinking history | 0.69 | 0.19-2.50 | 0.571 |
| Measurement days >4 | 1.55 | 0.34-7.03 | 0.572 |
| Heart rates | 1.03 | 0.97-1.08 | 0.174 |

CHD=Chronic heart disease.
