# Supplementary material for: Prevalence and Related Factors of White Coat Hypertension and Masked Hypertension in Shunde District, Southern China
Source: Front Physiol. 2022 Jul 1;13:936750. doi: 10.3389/fphys.2022.936750 (PMC9283833; doi:10.3389/fphys.2022.936750)
Supplement: Supplementary file 3 [file Table3.docx]

Table 3. Multivariate logistic regression analysis for related factors in Masked hypertension.

| Variables | OR | 95%CI | P |
| --- | --- | --- | --- |
| Male | 0.60 | 0.25-1.43 | 0.247 |
| Smoking history | 2.83 | 1.11-7.23 | 0.030 |
| Family history of hypertension | 2.17 | 1.11-4.26 | 0.024 |
| Family history of CHD | 2.82 | 1.07-7.45 | 0.036 |
| Exercise | 0.74 | 0.33-1.67 | 0.468 |
| Age | 1.01 | 0.97-1.04 | 0.337 |
| Overweight or obese | 1.77 | 0.89-3.53 | 0.104 |
| Drinking history | 0.79 | 0.36-1.75 | 0.557 |
| Measurement days >4 | 2.05 | 0.67-6.32 | 0.211 |
| Heart rates | 1.03 | 0.99-1.06 | 0.134 |

CHD=Chronic heart disease.
